# Multivariate analysis of polyphenolic content and in vitro antioxidant capacity of wild and cultivated berries from Bosnia and Herzegovina

**DOI:** 10.1038/s41598-021-98896-8

**Published:** 2021-09-28

**Authors:** Aleksandra Marjanovic, Jasmina Djedjibegovic, Aida Lugusic, Miroslav Sober, Luciano Saso

**Affiliations:** 1 Department of Pharmaceutical Analysis, Faculty of Pharmacy, University of Sarajevo, Zmaja od Bosne 8, 71 000 Sarajevo, Bosnia and Herzegovina; 2 Department of Physiology and Pharmacology “Vittorio Erspamer”, Sapienza University, P.le Aldo Moro 5, 00185 Rome, Italy

**Keywords:** Plant sciences, Chemistry

## Abstract

The aim of this study was to determine the antioxidant activity, total phenolics, total flavonoid, proanthocyanidins, and anthocyanins content of eight berry species, namely serviceberry, gooseberry, blackberry, black chokeberry, bilberry, red currant, black currant, and cornelian cherry harvested in the regions of Sarajevo and Tuzla, Bosnia and Herzegovina. The antioxidant activity was determined by a battery of in vitro tests including DPPH radical assay, FRAP assay, ABTS assay, and phosphomolybdate test for total antioxidant capacity. Total phenolics, total flavonoids, and proanthocyanidins ranged from 0.834 to 6.921 mg TAE (tannic acid equivalents), 0.081–0.673 mg of quercetin, and 0.162–3.247 mg of catechin per gram of fresh fruit, respectively. The water extract of fruits had considerable levels of tested constituents and antioxidant activity, with the highest results obtained for black chokeberry. The multivariate clustering analysis showed that water extracts of analyzed species of berries belong to four distinct types in terms of their antioxidants levels and antioxidant activity. Furthermore, these results support the opinion that employment of multiple antioxidant tests is indeed required for adequate in vitro assessment of antioxidant capacity. Results also emphasized the need for a more detailed evaluation of the fruit species with good antioxidant potential (relative to standards), such as cornelian cherry and gooseberry, which are abundant yet not frequently consumed in Bosnia and Herzegovina.

## Introduction

The oxidative stress theory of aging relies on the hypothesis that accumulation of the oxidative damage on cell macromolecules induced by reactive oxygen and nitrogen species (ROS and RNS) causes various age-related functional losses. Oxidative stress and cellular senescence are involved in the etiology of some chronic diseases, including diabetes, chronic inflammation, neurodegenerative disorders, and cancer^1–5^. A wide variety of plant antioxidants, phenolic compounds (phenolic acid, flavonoids, lignans, stilbenes, and tannins) play an important role in the metabolism of ROS and RNS^6^. A positive correlation between consumption of fruits, especially berries, and improvement of lipid profiles, enhancement of immune responses, and reduction of the oxidative damage on biomolecules, was reported in many studies and attributed to antioxidants present in this type of fruit^7,8^.

Berries commonly refer to small, pulpy fruits that belong to several families, including Rosaceae, Ericaceae, Grossulariaceae, and Corneaceae^8^. Berries are rich sources of bioactive compounds such as polyphenols (e.g.anthocyanins), vitamins A, C, E, and minerals. The content of nutritive and non-nutritive compounds (e.g., polyphenols) in fruits depends on cultivar and variety, cultivation systems, growing region, weather, and environmental conditions (e.g., temperature)^9–11^.

Berries from the family Grossulariaceae are native to the area with a moderate climate in North America but often cultivated in Bosnia and Herzegovina. More intensive production of black currant (*Ribes nigrum* L.), red currant (*Ribes rubrum* L.), and other berry fruits in Balkan countries begun at the second half of twentieth century, but only in relatively small production areas, while development of modern, intensive fruit production systems began at the end of the twentieth century and the beginning of the 21th century^12^ The most commonly cultivated and consumed species are red currant, black currant, and gooseberry (*Ribes uva-crispa* L.). Black chokeberry (*Aronia melanocarpa* (Michx.) Elliot) belongs to the family Rosaceae. Native to eastern North America, it is also intensively cultivated in Europe in the last century. It gains popularity in recent years since it is a rich source of many bioactive compounds with a wide range of health-promoting properties^13^. Cornelian cherry (*Cornus mas* L.) is widespread in Bosnia and Herzegovina, found mainly in the wild natural habitat. It has valuable nutritional properties, but despite that, it is not widely used and consumed^14^.

Serviceberry (*Amelanchier ovalis* Medik.) is native to Central and Southern Europe, North Africa, and the Middle East. This wild fruit can also be found in wild edible flora of Bosnia and Herzegovina^15^. It has a high content of phenolic compounds, as well as dietary fibers, vitamin B, potassium, and trace elements (cobalt and copper)^16^. Bilberry (*Vaccinium myrtillus* L.) is native to northern Europe but also found in parts of North America and Asia. Bilberry is especially rich in anthocyanins, but it also contains various other phenolic compounds (flavonols quercetin and catechins, condensed tannins and ellagitannins, phenolic acids), as well as a considerable amount of vitamin C^17^. Blackberry (*Rubus fruticosus* L.) is distributed throughout Europe, Asia, North and South America, and Oceania. It contains anthocyanins (predominantly cyanidin based in non-acylated form), phenolic acids, flavonoids, vitamins (A, C, and E), minerals (Zn, Cu, Al, Mn, and Fe), and carotenoids^18^.

The production of berries in Bosnia and Herzegovina highly expanded in recent years, especially the production of red raspberries (*Rubus idaeus* L.) and strawberries (*Fragaria* × *ananassa* Duch.)^19^. There are no official statistical data on the production of other soft fruit, namely berries. Some authors previously emphasized the importance of cultivars from the genus Ribes and Rubus and indicated the need for more intensive exploitation of these fruits^20,21^. Data on chemical analysis, especially phenolic compounds, and the antioxidative capacity of berries from Bosnia and Herzegovina are scarce.

Berries are often considered a „superfood “ due to their content of myriad bioactive compounds and generally high antioxidant capacity. However, such structural complexity can be very challenging from the analytical aspect and species differentiation in terms of their relative nutritive and medical quality. As a solution, the use of the chemometric approach has been increasing recently. For example, the multivariate analysis was previously used: to successfully differentiate novel strawberry cultivars^22^, for grouping of plant extract based on their antioxidant activities^23^, to assess the solvent effect on antioxidants extraction and antioxidant activity of the berries extract^24^, classification of certain edible and medicinal plants in terms of their antioxidant capacity, toxicity and antimycobacterial power^25^, and to assess the sensitivity of antioxidant assays in the various plant extract^26^.

Thus, the aim of this study was to investigate polyphenolic content and antioxidant capacity of selected cultivated (red currant, black currant, gooseberry, black chokeberry, and blackberry) and wild (bilberry, serviceberry, and cornelian cherry) small soft fruits harvested in the region of Sarajevo and Tuzla, Bosnia and Herzegovina. Additionally, it was examined whether these species show a tendency to group based on the similarity in the content of phenolic components and the estimated antioxidant capacity.

## Results

### Polyphenolics content

The concentration of phenolic compounds in analyzed samples are presented in Table 1.

**Table 1:** Content of phenolic compounds in samples expressed on wet weight.

Sample	Total phenolics (TAE mg/g)	Total flavonoids (QE mg/g)	Proanthocyanidins (CE mg/g)	Anthocyanins (C3GE mg/100 g)
Black chokeberry	6.921 ± 0.011^a^	0.673 ± 0.001^a^	0.162 ± 0.022^a^	22.08 ± 3.25^a, b^
Serviceberry	2.074 ± 0.017^b^	0.214 ± 0.003^b^	1.057 ± 0.026^b^	26.05 ± 1.66^a^
Red currant	1.855 ± 0.003^c^	0.081 ± 0.000^c^	1.484 ± 0.004^c^	4.630 ± 0.39 ^a,b^
Black Currant	2.286 ± 0.004^d^	0.113 ± 0.001^d^	1.536 ± 0.009^d^	24.55 ± 3.82^a^
Gooseberry	1.223 ± 0.013^e^	0.091 ± 0.001^e^	1.286 ± 0.007^e^	0.080 ± 0.01^b^
Bilberry	4.169 ± 0.009f.	0.321 ± 0.001f.	3.115 ± 0.011f.	20.35 ± 3.41^a^
Cornelian cherry	3.623 ± 0.002^g^	0.100 ± 0.000^g^	2.403 ± 0.002^g^	80.57 ± 8.43^c^
Blackberry	2.619 ± 0.018^h^	0.242 ± 0.001^h^	3.247 ± 0.024^h^	198.9 ± 19.5^d^

TAE – tannic acid equivalents; QE – quercetin equivalents; CE – catechin equivalents; C3GE—cyanidin-3-glucoside equivalents; Means not sharing the same letter in the same column are significantly different at *p* < 0.05 probability.

### Antioxidant capacity assessed by different tests

The results of four in vitro tests (DPPH, TAC, FRAP, and ABTS) for the evaluation of antioxidant capacity are summarized in Table 2.

**Table 2:** Antioxidant activity of samples determined by DPPH, TAC, FRAP, and ABTS assays (all results expressed on wet weight).

Sample	DPPH		TAC	FRAP	FRAP	ABTS
	% inhibition	EC_50_ (v/v %)	AA mg/g	Fe^2+^ mmol/kg	Fe^2+^ mmol/L	Trolox mmol/kg
Black chokeberry	87.68 ± 2.09^b^	0.04 ± 0.00^a^	1.063 ± 0.035f.	42.54 ± 3.83^d^	85.08 ± 7.66	80.89 ± 6.35^b^
Serviceberry	34.35 ± 1.22^c^	2.24 ± 0.24^b^	0.488 ± 0.018^c^	0.10 ± 0.01^e^	0.20 ± 0.02	45.98 ± 4.14^a^
Red currant	75.42 ± 2.88^a^	0.20 ± 0.02^a^	0.712 ± 0.025^a,b^	26.97 ± 1.61^a,c^	53.94 ± 3.22	77.02 ± 3.95^b^
Black currant	72.23 ± 2.82^a^	0.22 ± 0.03^a^	1.403 ± 0.085^e^	27.79 ± 2.39^a,c^	55.58 ± 4.78	76.87 ± 6.37^b^
Gooseberry	45.10 ± 1.60^d^	0.23 ± 0.02^a^	0.621 ± 0.020^b,c^	19.44 ± 0.88^b^	38.88 ± 1.76	54.53 ± 4.51^a,c,e^
Bilberry	85.89 ± 1.34^b^	0.12 ± 0.01^a^	0.837 ± 0.060^a^	32.38 ± 1.76^c^	64.76 ± 3.52	65.79 ± 7.05^b,c^
Cornelian cherry	77.57 ± 2.14^a^	0.11 ± 0.01^a^	2.018 ± 0.110^d^	39.97 ± 3.03^d^	79.94 ± 6.06	60.45 ± 5.07^a,c,d^
Blackberry	77.76 ± 1.40^a^	0.21 ± 0.02^a^	0.712 ± 0.021^a,b^	24.71 ± 1.97^a,b^	49.42 ± 3.94	66.00 ± 3.75^b,d,e^
AA	34.35 ± 0.66				24.30	
Catechin	70.17 ± 3.01				27.87	
Trolox	39.17 ± 1.80	1.93 ± 0.09			28.26	

AA-ascorbic acid; Means not sharing the same letter in the same column are significantly different at *p* < 0.05 probability.

Concentrations for standards AA, catechin and Trolox were 50 μmol/L.

### Correlation between test results

The results of the Pearson's correlation between the individual antioxidant assays results and also between phenolics content and the antioxidant assays data are presented in Table 3.

**Table 3:** Pearson's correlation coefficients (r) and the corresponding *p* values for different antioxidative capacity assays and the Pearson's correlation between the phenolics content and the antioxidant assays data (N = 24).

		DPPH % inhibition	DPPH EC_50_ (v/v %)	TAC AA mg/g	FRAP Fe^2+^ mmol/kg	
DPPH	r	− 0.772**				
EC_50_ (v/v %)	*p*	< 0.000				
TAC	r	0.453*	− 0.420*			
AA mg/g	*p*	0.026	0.041			
FRAP	r	0.884**	− 0.848**	0.658**		
Fe^2+^ mmol/kg	*p*	< 0.000	< 0.000	< 0.000		
ABTS	r	0.764**	− 0.648**	0.265	0.688**	
Trolox mmol/kg	*p*	< 0.000	0.001	0.211	< 0.000	

		DPPH % inhibition	DPPH EC_50_ (v/v %)	TAC AA mg/g	FRAP Fe^2+^ mmol/kg	ABTS Trolox mmol/kg
Total phenolics	r	0.648**	− 0.310	0.310	0.673**	0.447*
(TAE mg/g)	*p*	0.001	0.141	0.141	< 0.001	0.029
Total flavonoids	r	0.421*	− 0.101	− 0.093	0.379	0.350
(QE mg/g)	*p*	0.041	0.639	0.655	0.068	0.094
Proanthocyanidins	r	0.311	− 0.255	0.106	0.097	− 0.143
(CE mg/g)	*p*	0.139	0.229	0.621	0.651	0.504
Anthocyanins	r	0.243	− 0.118	0.103	0.071	− 0.059
(C3GE mg/100 g)	*p*	0.253	0.583	0.631	0.741	0.785

*Correlation is significant at the 0.01 level (2-tailed).

**Correlation is significant at the 0.05 level (2-tailed); “r-Pearson’s coefficient of correlation, *p* probability.

### Berries classification by multivariate analysis

**Figure 1:**
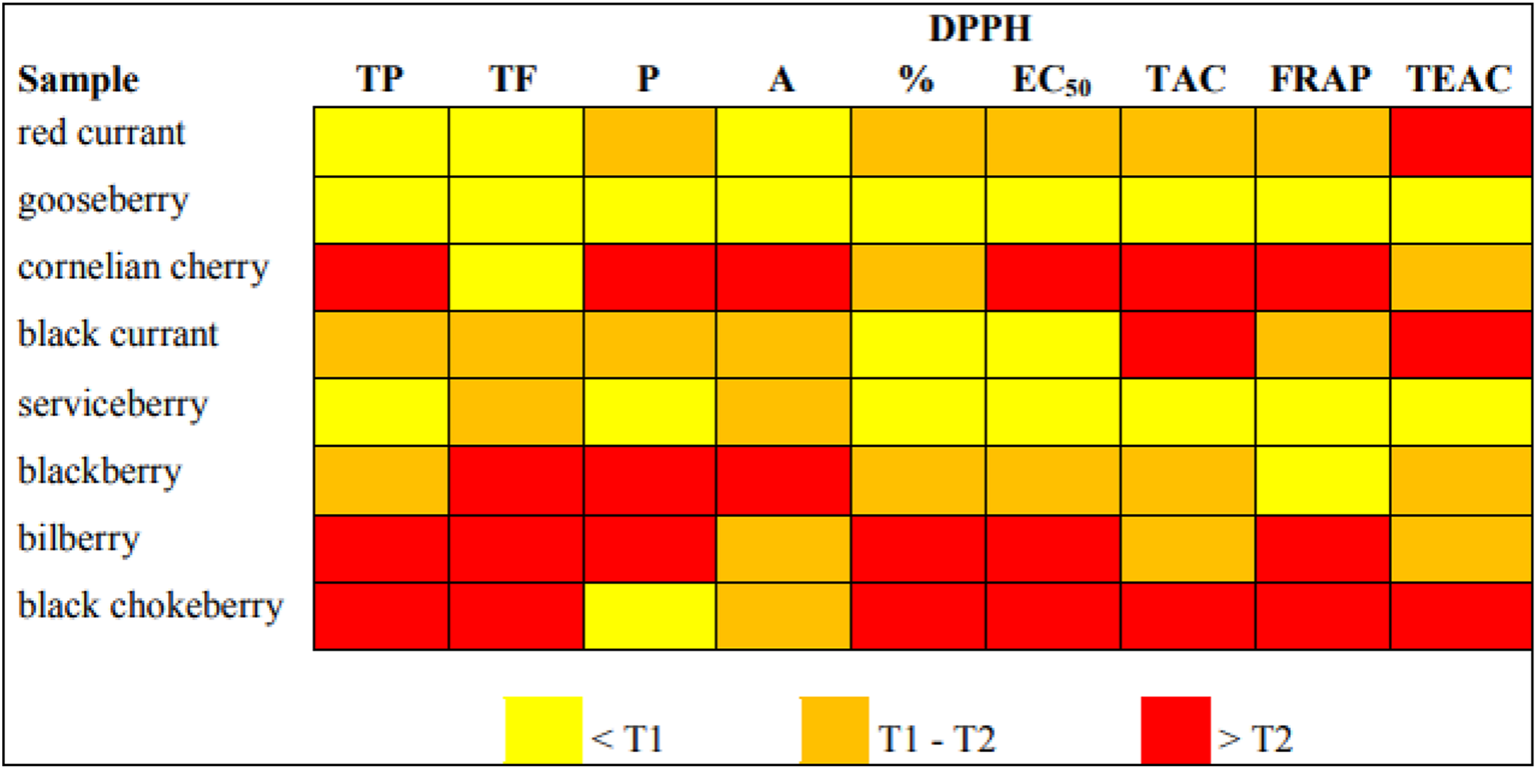
Classification of berries into tertiles by test results (in reverse order for DPPH EC_50_). *TP* total phenolic compounds, *TF* total flavonoids, *P* proanthocyanidins, *A* anthocyanins; *T1* first tertile, *T2* second tertile.

The variability of different species across the conducted tests is presented in Fig. 1.

**Figure 2:**
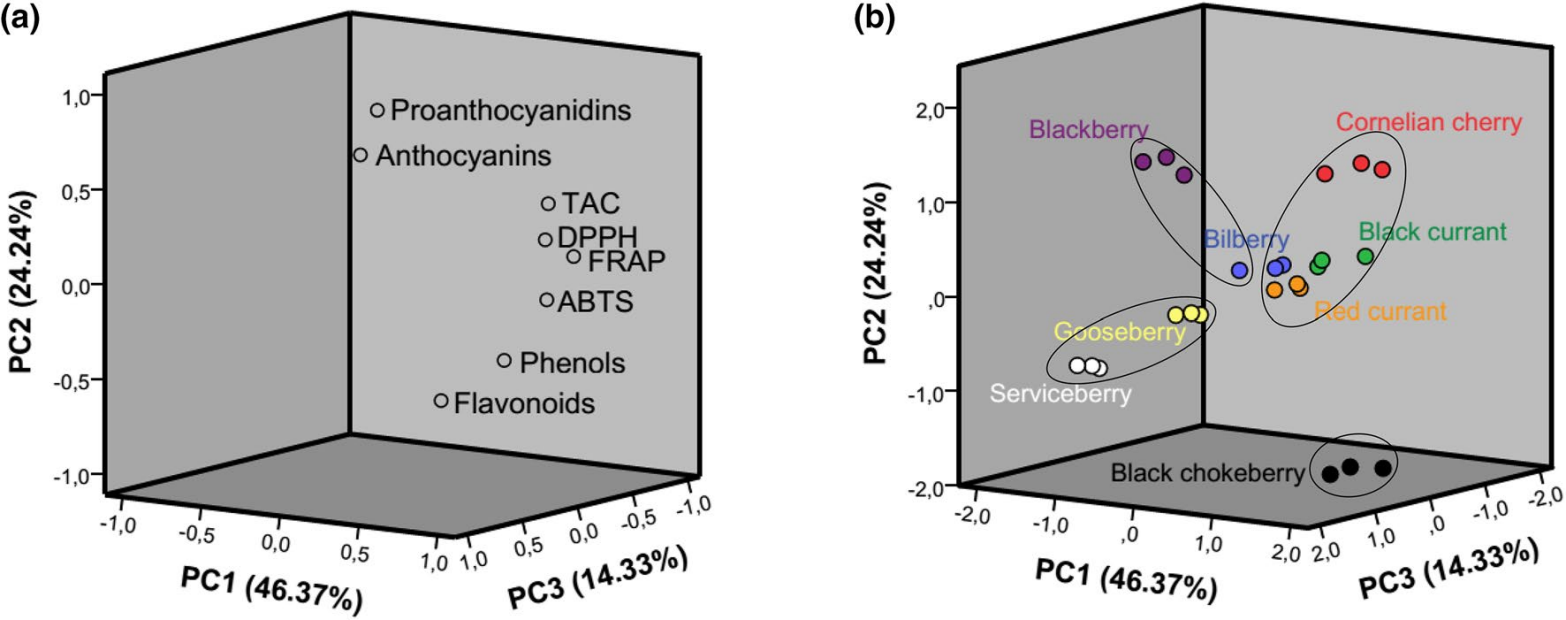
(**a**) Factors loading plot on PC1xPC2xPC3 and (**b**) scores plot of samples.

The results of principal component analysis (PCA) of the data are presented in Fig. 2. and Table 4.

**Table 4:** Variable loadings, eigenvalues and percentage of cumulative variance for the first three principal components (PC).

Variable	PC1	PC2	PC3
Total phenolics	0.849	− 0.321	0.290
Total flavonoids	0.635	− 0.517	0.557
Proanthocyanidins	− 0.004	0.902	0.222
Anthocyanins	0.109	0.723	0.538
DPPH	0.919	0.281	0.018
TAC	0.533	0.349	− 0.560
FRAP	0.926	0.158	− 0.229
ABTS	0.766	− 0.085	− 0.214
Eigenvalue	3.709	1.939	1.147
% of variance	46.37	24.24	14.33

The agglomeration coefficients plot generated by the hierarchical cluster analysis of the PC scores is presented in Supplementary material (Fig. S6).

Results of the non-hierarchical K-means clustering of the PCA scores are presented in Table 5.

**Table 5:** Final cluster centers, species in clusters, and profiles description.

Factor score	Cluster				Profile description
	1	2	3	4	
1	− 1.393	0.262	0.080	1.721	Total phenols- and flavonoids-rich, broad reactivity antioxidant
2	− 0.681	0.375	1.236	− 1.661	Proanthocyanidin- and anthocyanins-Dominated, TAC-responding antioxidant
3	0.127	− 0.770	1.434	0.657	Flavonoids-rich, anthocyanins-dominated, TAC non-responding antixidant
Species	Gooseberry, serviceberry	Red currant, black currant, cornelian chery, bilbery*	Blackberry, bilbery*	Black chokeberry	

*Bilberry samples split into two clusters.

## Discussion

Many studies use organic solvents to enhance the extraction of polyphenolic substances from plant material. While this is an effective approach, it leads to higher bioaccessibility very unlikely to be achieved by a common way of consumption. As previously highlighted by some authors, the presence of water increases the permeability of cell tissue, thus resulting in better mass transfer by molecular diffusion and better recovery of water-soluble bioactive compounds^24^. Therefore, we used the water extracts of berries which better reflects the most probable consumption scenario.

The total phenolics ranged from 1.223–6.921 tannic acid equivalents (TAE mg/g w.w) (Table 1). Once considered as antinutrients, polyphenolic compounds, are nowadays recognized as important bioactives with many health-protective properties, namely antioxidative, antimicrobial, anti-inflammatory, hepatoprotective, and anticarcinogenic^27^.

Total flavonoids content, expressed as quercetin equivalents, varied from 0.081 QE mg/g w.w. in red currant to 0.673 QE mg/g w.w. in black chokeberry. The average content of total flavonoids was higher in our samples (Table 1) than the content previously reported for the same fruit species (e.g., 16.05 QE mg/100 g in serviceberry or 4.48 mg/100 g in black currant)^28^. Proanthocyanidins expressed as catechin equivalents ranged from 0.162 CE mg/g w.w. in black chokeberry to 3.247 CE mg/g w.w. in blackberry. The average content of proanthocyanidins in our samples was comparable with those previously reported^29–31^.

The highest anthocyanins content (198.9 C3GE mg/100 g w.w.) was found in blackberry (Table 1). The anthocyanin content of six different blackberry cultivars reported by Cho et al. was in the range 85.2–190.6 C3GE mg/100 g w.w.^32^, which is slightly lower than the content measured in our sample. The lowest anthocyanins content was in the gooseberry sample (0.08 C3GE mg/100 g w.w.). Wu et al. recorded anthocyanin concentrations in four different cultivars of gooseberry ranging from 0.05 to 5.42 C3GE mg/100 g w.w.^33^. The average content of anthocyanins in chokeberry, black currant, serviceberry, and bilberry was 23.26 mg C3GE mg/100 g w.w., which is similar to data reported by other authors^32–37^.

The total anthocyanin content in cornelian cherry (80.57 C3GE mg/100 g w.w.) was in good agreement with results obtained by Bijelic et al. for samples from Serbia^38^. However, higher anthocyanins content was reported for samples from Iran (192.7 mg/100 g w.w.)^39^. Similarly, anthocyanin content in the range from 389.10 ± 3.81 and 398.92 ± 1.79 C3GE mg/100 g w.w was reported by Islamovic et al. for cornelian cherry samples collected from Konjic and Bugojno (Bosnia and Herzegovina) in 2012 and 2013^40^. Total anthocyanins in bilberry samples collected in Bosnia and Herzegovina (Konjic, Busovača, and Fojnica) in 2007 ranged from 2.7 to 4.5 C3GE mg/g w.w^41^, thus being more than tenfold higher than in our sample. However, the content reported in the same study for cultivated blackberry collected in Cazin (0.7–1.0 C3GE mg/g w.w) was lower than what we found for the same species. This finding confirms that wild edible plants, especially fruits, are rich in bioactive compounds compared to cultivated crops as ordinary food.

All investigated samples showed good antioxidant properties, as compared to numerous other foods (fruits, vegetables, beverages and vegetable oils), reported by other authors^42–44^.

The results of the DPPH test showed the highest antioxidant capacity for black chokeberry (87.68% inhibition) with the lowest EC_50_ (0.04 v/v%) (Table 2). The highest effective concentration (EC_50_ = 2.24 v/v%) was obtained for serviceberry, which correlates well with the DPPH % of inhibition (34.35%). The effective concentration (EC_50_) for all analyzed samples except for serviceberry was lower than for Trolox, used as pure standard (Table 2). Similarly, those samples showed higher antioxidant capacity (% inhibition) comparing to ascorbic acid and Trolox. The antioxidant capacity of chokeberry, red currant, black currant, bilberry, cornelian cherry, and blackberry were even higher than that of catechin (% of inhibition = 70.17).

Cornelian cherry showed the highest TAC value (2.018 mg AA/g w.w.), slightly higher than that reported by Hassanpour et al.^38^. Strong antioxidant capacity in this test was also recorded for black chokeberry, followed by black currant, whereas serviceberry exhibited the lowest values (Table 2).

FRAP results in our study were comparable with those previously reported for the red currant, black currant and bilberry^43^, black chokeberry^44^, and gooseberry^45^. FRAP values for the blackberry and red currant were lower, while our bilberry had almost twice higher value than reported by Pellegrini et al.^42^. In the FRAP assay, all investigated samples, except serviceberry, exhibited higher antioxidant capacity relative to pure, standard antioxidants (ascorbic acid, catechin, and Trolox).

The highest ABTS value was recorded for black chokeberry, followed by red currant and black currant (Table 2). ABTS values reported for the blackberry, bilberry, and red currant in our study are slightly higher than those reported by Pellegrini et al.^42^. Generally, such variations might be due to different environmental conditions, harvesting time and stage of fruit ripeness, genetic differences among varieties, as well as extraction and testing methods.

Furthermore, the sample of cornelian cherry analyzed in our study exhibited good antioxidant capacity in all in vitro tests (DPPH, total antioxidant activity, FRAP, and ABTS), comparable with the standard antioxidants.

Significant correlations were found between individual antioxidant assays results (Table 3). The only exception here was an insignificant correlation between TAC and ABTS results. TAC assay also showed a somewhat weaker correlation to the DPPH assays in this study, while a high correlation was found with the FRAP assay. Furthermore, the TAC assay had the lowest correlation coefficients (r = 0.265–0.658) relative to other assays (r = 0.648–0.884).

While good correlations are commonly reported between DPPH, ABTS, and FRAP assays, the correlation between TAC and other antioxidant assays remains debatable. The latter could be due to distinct reaction mechanisms of the tests (electron transfer—ET or hydrogen atom transfer—HAT). The FRAP and TAC are ET assays, while DPPH and ABTS measure both ET and HAT reactions. Therefore, a good correlation between DPPH and ABTS could be expected. FRAP assay is sensitive and not very specific, so it often correlates well with DPPH and ABTS. Based on the reaction mechanism it could be expected to also correlate with TAC assay, but the reported data here is conflicting^46^.

Several authors previously noted that DPPH and ABTS assays respond to antioxidants such as polyphenolics including flavonoids and phenols while phosphomolybdenum TAC assay responds well to certain antioxidants, namely ascorbic acid, some phenolics, α-tocopherol, and carotenoids^46^. This finding may also explain why high polyphenolic content does not reflect a significant TAC in the phosphomolybdenum TAC assay. In contrast, DPPH and ABTS usually show a good correlation with total phenolics and/or flavonoids^46^. Indeed, our results clearly show this difference since the TAC assay was the only of the four tests which did not show a good correlation with total phenolics in our samples (Table 3). Significant positive correlation was also found for total flavonoids vs. total phenols (r = 0.900, *p* < 0.001) and proanthocyanidins vs. anthocyanins (r = 0.618, *p* = 0.001). Other correlations between the analyzed compounds were not significant.

The variability of different species across the conducted tests is visually depicted in Fig. 1. Although very simplified, this presentation suggests certain similarities (for example DPPH and FRAP results for black chokeberry and bilberry both fall into third tertile, i.e. > T2), but also some discrepancies (for example in the total proanthocyanidins content, with black chokeberry falling in first tertile i.e. < T1, and bilberry falling in the third tertile, i.e. > T2). To better understand the underlying relationships and their meaning in the context of species classification, we further analyzed the data by principal component analysis (PCA) and clustering analysis. The used PCA model suggested three principal components (Fig. 2a), accounting for 84.94% of the total variation (PC1 46.37%, PC2 24.24%, PC3 14.33%).

As can be observed from the factors loadings plot (Fig. 2a) and the corresponding eigenvalues (Table 4), variables are grouped in three groups, namely (1) proanthocyanidins and anthocyanins, (2) antioxidant assays (TAC, DPPH, FRAP, and ABTS), and (3) total flavonoids and to some extent total phenolic compounds. The grouping based on a positive correlation between the variables is in good agreement with Pearson's correlation shown in Table 3. Interpreting the meaning of the PCs, we could say that PC1 is characterized by a high (> 0.3) positive load of antioxidant assays (DPPH, FRAP, ABTS, and TAC to a lesser extent), as well as total phenolic compounds and flavonoids. Thus, the PC1 profile could be described as „total phenols- and flavonoids-rich, broad reactivity antioxidant “. In the same way, PC2 profile can be described as „proanthocyanidin- and anthocyanins-dominated, TAC-responding antioxidant “, and PC3 profile as ‘’flavonoids-rich, anthocyanins-dominated, TAC non-responding weak antioxidant “. The plot of objects (i.e., samples) scores (Fig. 2b) suggests at least four distinct groups, with the replicates for each species clustered tightly together. To finally determine the number of groups (clusters), we analyzed the PC scores (for the first three PCs) by the hierarchical cluster analysis using Ward's method based on squared Euclidean distance. By inspection of the resulting agglomeration coefficients table (Table S1 in Supplementary material) and agglomeration plot (Fig. S6 in Supplementary material) and using the „elbow rule “ we concluded that the definite number of clusters is four (24–20 = 4). To confirm samples' cluster membership, we further conducted the non-hierarchical K-means clustering of the PCA scores with the defined number of clusters (four). The results (Table S2 in Supplementary material) confirmed the clustering presented in Fig. 2b. All the samples from the same species were clustered together, except the bilberry. Bilberry samples fall on the borderline of two clusters (clasters 2 and 3), indicating that they share certain similarites with each of these distinct clusters. Finally, to assess how these clusters relate to the previously described PC profiles, we looked at the final cluster centers generated by K-means clustering (Table 5). Bearing in mind that higher positive values mean a higher similarity and higher negative values higher dissimilarity, we can conclude the following:

- Cluster 1 is very far from profiles 1 and 2 and not very similar to profile 3
- Cluster 2 is very far from profile 3 and more similar to profile 2
- Cluster 3 is extremely similar to profiles 2 and 3
- Cluster 4 is extremely similar to profile 1 and very far from profile 2.

## Conclusions

Samples analyzed in this study contained a substantial amount of phenolic compounds and showed variable antioxidant capacity in a battery of in vitro tests. The multivariate clustering analysis showed that water extracts of analyzed species of berries belong to four distinct types according to their antioxidants levels and antioxidant activity. Red currant, black currant, and cornelian cherry responded better in TAC than in the FRAP, DPPH, and ABTS. Thus, it is recommendable to include the TAC assay when water extract of these species is tested. These results should be additionally confirmed in more samples and different concentration ranges. Furthermore, the results support the opinion that the employment of multiple antioxidant tests is not a redundancy but rather a necessity in the assessment of the antioxidant capacity of a specific matrix.

The results also emphasized a need for a more detailed evaluation of some fruit species, such as cornelian cherry and gooseberry, which are underutilized in Bosnia and Herzegovina despite their antioxidant potentials. Although serviceberry had the lowest values in all used assays, still those results are comparable with the standard antioxidants. Bearing in mind that serviceberry is widespread in wild edible flora of Bosnia and Herzegovina, its nutritive and biomedical potential should not be neglected.

## Materials and methods

### Location and sample collection

Eight different types of berries were used in this study. Fruits were collected at the private land (with consent and permission of owners) in the region of Sarajevo (43° 50′ 55.10" N, 18° 21′ 23.18" E) and Tuzla (44°32′18.31" N, 18°40′1.52" E) during July–September 2019. The region is characterized by moderate continental climate. The average temperature in Sarajevo during sampling season was 17.1** °C**, approximately 5** °C** higher than the average for 2019. Humidity was 70% (3% higher than average for 2019), and precipitation was 60.5 mm (70.9 mm average for 2019). In Tuzla region, average temperature was 19.9** °C** (12.2** °C** average for 2019). Humidity was 82% (80% average for 2019), and precipitation was 67.8 mm (74.2 mm average for 2019)^47^.

Black currant (cultivar “Cacanska crna”), red currant (cultivar “Rondom”), and gooseberry (cultivar “Gelbe triumph”) were from Sarajevo, while black chokeberry (cultivar “Nero”) and blackberry (cultivar “Jumbo”) were from Tuzla. Cultivated species were grown in traditional home gardens. Formal identification of the wild fruits analyzed in this study (bilberry, serviceberry, and cornelian cherry, from the Tuzla region), was performed by professor, Kemal Duric, PhD (Department of Pharmacognosy, Faculty of Pharmacy, University of Sarajevo, BiH). Voucher specimens for bilberry (number 0056/19), serviceberry (number 0057/19) and cornelian cherry (number 0058/19) were deposited at the herbarium of the Department of Pharmacognosy, Faculty of Pharmacy, University of Sarajevo. Collecting and handling with the plant material (either cultivated or wild) was performed in accordance with all the relevant institutional, national, and international guidelines and legislation.

The samples were collected from 3 to 10 individual plants, about 100 g of each species. Samples were transported and stored at refrigerator temperature (~ 4 °C) and analyzed within 24 h at the laboratory of the Faculty of Pharmacy in Sarajevo.

### Chemicals and sample preparation

All the chemicals and reagents used were of analytical grade and purchased from Sigma Aldrich (St.Louis, MO, USA) and Merck (Germany).

For the extraction, 5.000 g of fruit was homogenized in 25 ml of distilled water, and the mixture was sonicated for 30 min at 20 ± 2 °C. Extracts were then filtered through filter paper (Whatman no.1) and used for further analysis. The spectrophotometric measurements were performed on a Shimadzu UV-1280 spectrophotometer. All the samples were analyzed in triplicate.

### Total phenolic content

Total phenolic content was determined using the Folin-Ciocalteu spectrophotometric method^48^. In brief, 0.1 mL of the Folin-Ciocalteu reagent and 1.58 mL of distilled water were added to test aliquots (20 μL). After 8 min, 0.3 mL of aqueous sodium bicarbonate solution (20%) was added and thermostated for 30 min at 40 °C. The absorbance was measured at 765 nm, and the content of total phenolic was calculated from the calibration curve (presented in Supplementary material, Fig. S1) prepared with tannic acid standard (range 100–1000 mg/L). The results were expressed as mg TAE per g of wet weight.

### Total flavonoid content

The total flavonoid content was estimated by using the slightly modified colorimetric method originally described by Woisky and Salatin^49^ and applicable for glycosylated or non-glycosylated flavonoids determination^50^. An aliquot (1.5 mL) of the extract was mixed with 3 mL of methanol, 0.2 mL of 10% aluminum chloride, 0.2 mL of 1 M potassium acetate, and 5.6 mL of distilled water. The mixture was incubated for 30 min at room temperature before the absorbance was measured at 415 nm. A standard solution of quercetin (range 10–250 mg/L) was used for the construction of the calibration curve presented in Supplementary material (Fig. S2). The results were expressed as mg QE per g of wet weight.

### The proanthocyanidin content

The proanthocyanidin content was determined by the vanillin-HCl method described by Sun et al.^51^ and expressed as catechin (hydroxy flavan-3-ol) equivalents. This colorimetric method is specific for the condensed tannins. It is based on the reaction of vanillin with m-substitued A ring of flavanol, leading to a formation of a chromophore whose concentration is proportional to the absorbance measured at 500 nm. Calibration curve used for the calculation of the proanthocyanidin content was prepared in a way described in more detail in Supplementary material (Fig. S3). The results were expressed as mg CE per g of wet weight.

### Total anthocyanins content

For the determination of total monomeric anthocyanins, the pH-differential method was used^52^. Two aliquots (1 mL) of extract were mixed with 3 ml of either 0.025 M potassium chloride (pH = 1.0) or 0.4 M sodium acetate (pH = 4.5). After incubation of 15 min at room temperature, absorbance was measured at two wavelengths (λ = 520 and λ = 700 nm). For the calculation of the content of anthocyanins (expressed as cyanidin-3-glucoside equivalents per 100 g of wet weight ) equation presented in Supplementary material was used.

### DPPH (2,2-diphenyl-1-picrylhydrazyl) assay

The scavenging activity was measured with DPPH (2,2-diphenyl-1-picrylhydrazyl) assay, as previously described^53^. In brief, 50 μL of the extract was mixed with 2 mL of ethanolic solution of DPPH (20 μM). The absorbance was measured after 960 s at 517 nm. The percentage of the inhibition of DPPH radicals was calculated using equation described in Supplementary material. Catechin, ascorbic acid, and Trolox (6-hydroxy-2,5,7,8-tetramethylchroman-2-carboxylic acid) were used as pure standards (1 mL of 50 μM solutions). For all the analyzed samples and Trolox, the effective concentrations necessary to scavenge 50% of DPPH radicals (EC_50_) were calculated using graphical regression analysis and expressed as v/v % (relative to the volume of DPPH solution).

### FRAP (ferric-reducing antioxidant power) assay

Antioxidant capacity was also tested using FRAP (Ferric-Reducing Antioxidant Power) assay^53,54^. A 50 µL of the extract was mixed with 1.5 mL of FRAP reagent prepared by mixing 10 mL of 300 mM acetic buffer pH 3.6 with 1 mL of 10 mM TPTZ (2,4,6-tripyridyl-s-triazine) in 40 mM hydrochloric acid and 1 mL of 20 mM solution of FeCl_3_ × 6 H_2_O just before measurement of the absorbance at 593 nm. For the preparation of the calibration curve (Fig. S4 in Supplementary material) standard solution of the Fe (II)SO_4_ × 7 H_2_O (250–3500 µM) was used. Results were expressed in mmol Fe^2+^ per L and mmol/kg Fe^2+^ (wet weight). Ascorbic acid, catechin, and Trolox were used as pure standards.

### Total antioxidant capacity (TAC)

Total antioxidant capacity was determined using the method previously described by Prieto et al.^55^. A 3 mL of reagent solution (0.6 M sulphuric acid, 28 mM sodium phosphate, and 4 mM ammonium molybdate) was added to a 0.3 mL of sample, incubated for 90 min at 95 ^0^C, and measured at 695 nm, against the blank (0.3 mL of methanol with 3 mL of reagent solution). Ascorbic acid (range 10–500 mg/L) was used as the calibration standard. The calibration curve is shown in Supplementary material (Fig. S5). The results were expressed as mg AA per g of wet weight.

#### Total antioxidant potential test (ABTS)

Total antioxidant potential test (ABTS) was performed according to the procedure previously described by Re et al.^56^. Equal volumes of 7 mM solution of ABTS (2,2'-azino-bis (3-ethylbenzothiazoline-6-sulphonic acid) and 2.45 mM solution of K_2_S_2_O_8_ (oxidant) were mixed and kept in dark for 14 h before the use. The working solution was diluted with ethanol to adjust absorbance to 0.7 ± 0.02 (λ = 734 nm). The standard ethanolic solution of Trolox was used for the calibration curve (range 0–15 μM). An aliquot of 0.5 mL of each standard or 0.02 mL of extract was mixed with 1 mL of ABTS solution, and the absorbance was measured every 10 s for 15 min in total. ABTS values expressed as Trolox equivalent (TE, μmol/L Trolox) were calculated using equations presented in Supplementary material and the results were reported as mmol TE per kg of wet weight.

#### Statistical analysis

All the samples were analyzed in triplicate, and the results are expressed as mean ± standard deviation. Significant differences in antioxidant activity and phenolic compounds content in different samples were determined using one-way ANOVA with Bonferroni post-hoc test. The correlation coefficients were calculated with Pearson’s test. The principal component analysis (PCA) and cluster analysis were used to explore sample similarities and grouping according to their content of phenolic compounds and antioxidant capacity. Data clustering is often ambiguous and is not easily validated. To minimize the possibility of inaccurate determination of clusters, we first conducted the PCA analysis on standardized data. The DPPH EC_50_ was excluded to avoid redundancy and because it showed a lower correlation with other tests comparing to DPPH % inhibition. Thus, the data matrix contained 24 objects (eight species analyzed in triplicate) and eight variables (total phenolics, flavonoids, proanthocyanidins, anthocyanins, DPPH % inhibition, TAC, FRAP, and ABTS). The extraction criterion was eigenvalue > 1. Statistical significance was established at *p* < 0.05. Data analysis was performed using the IBM SPSS Statistics V23.0 software.

## Supplementary Information

Supplementary Information.

## Data Availability

The datasets generated during and/or analysed during the current study are available from the corresponding author on reasonable request.
