# Supplementary material for: Multivariate analysis of polyphenolic content and in vitro antioxidant capacity of wild and cultivated berries from Bosnia and Herzegovina
Source: Sci Rep. 2021 Sep 28;11:19259. doi: 10.1038/s41598-021-98896-8 (PMC8478928; doi:10.1038/s41598-021-98896-8)
Supplement: Supplementary file 1 — Supplementary Information. [file 41598_2021_98896_MOESM1_ESM.pdf]

Supplementary material

Multivariate analysis of polyphenolic content and in vitro antioxidant capacity of wild and cultivated berries from Bosnia and Herzegovina

Aleksandra Marjanovic [1,*], Jasmina Djedjibegovic [1], Aida Lugusic [1], Miroslav Sober [1] and Luciano Saso [2]

[1]University of Sarajevo, Faculty of Pharmacy, Department of Pharmaceutical analysis, Zmaja od Bosne 8, 71 000 Sarajevo, BiH

[2]"Department of Physiology and Pharmacology "Vittorio Erspamer", Sapienza University P.le Aldo Moro 5, 00185, Rome, Italy Tel & Fax 0039-06-49912481

[*]Correspondence: aca1902@gmail.com

Table of contents:

Figure S1. Calibration curve for the determination of the total phenolic content

Figure S2. Calibration curve for the determination of the total flavonoid content

The proanthocyanidin content calculation

Figure S3. Calibration curve for the determination of the proanthocyanidin content

Total anthocyanins content calculation

DPPH (2,2-Diphenyl-1-picrylhydrazyl) assay calculation

Figure S4. Calibration curve for the FRAP assay

Figure S5. Calibration curve for the determination of total antioxidant capacity (TAC)

TEAC values calculation

Figure S6. The plot of agglomeration coefficients generated by Ward's hierarchical clustering analysis

Table S1. Agglomeration schedule table generated by the Ward's chierarchical clustering analysis

Table S2. Cluster membership determined by *k*-means clustering analysis

Total phenolic content

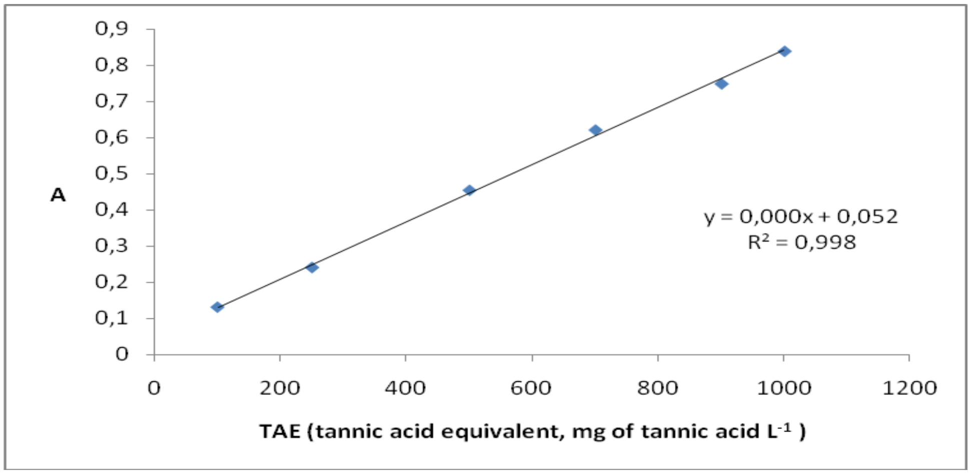

SUMMARY OUTPUT

Regression Statistics	
Multiple R	0,99924983
R Square	0,99850023
Adjusted R Square	0,99812529
Standard Error	0,01221581
Observations	6

ANOVA

	df	*SS*	*MS*	*F*	*Significance F*
Regression	1	0,397400596	0,397401	2663,078	8,43912E-07
Residual	4	0,000596904	0,000149		
Total	5	0,3979975			

	Coefficients	*Standard Error*	*t Stat*	*P-value*	*Lower 95%*	*Upper 95%*
Intercept	0,0519589	0,010105061	5,141869	0,006782	0,023902756	0,0800151
X Variable 1	0,00078877	1,52847E-05	51,60502	8,44E-07	0,00074633	0,0008312

Figure S1. Calibration curve for the determination of the total phenolic content

Total flavonoid content

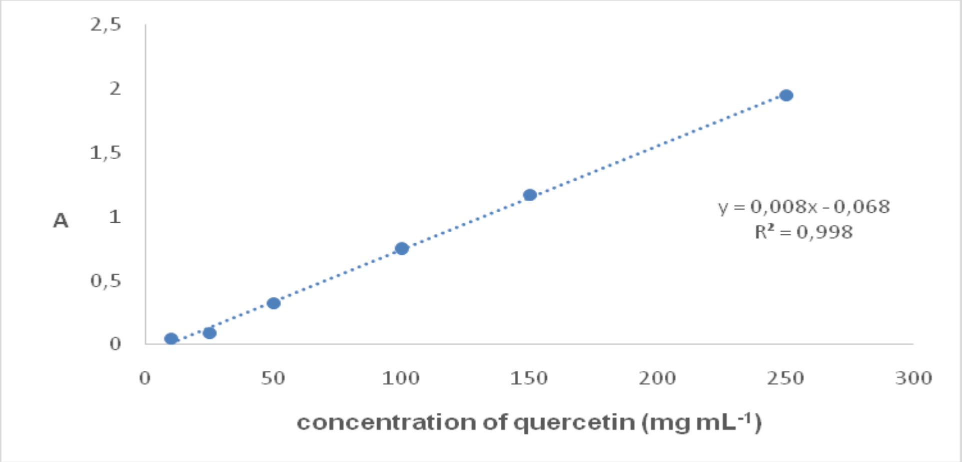

SUMMARY OUTPUT						
Regression Statistics						
Multiple R	0,99924963					
R Square	0,99849983					
Adjusted R Square	0,99812479					
Standard Error	0,03184061					
Observations	6					
ANOVA						
	df	*SS*	*MS*	*F*	*Significance F*	
Regression	1	2,699174036	2,699174	2662,369	8,44361E-07	
Residual	4	0,004055297	0,001014			
Total	5	2,703229333				
	Coefficients	*Standard Error*	*t Stat*	*P-value*	*Lower 95%*	*Upper 95%*
Intercept	-0,06862468	0,020074004	-3,41858	0,026815	-0,12435905	-0,0128903
X Variable 1	0,0080953	0,000156891	51,59815	8,44E-07	0,007659696	0,0085309

Figure S2. Calibration curve for the determination of the total flavonoid content

The proanthocyanidin content calculation and calibration curve

For the preparation of the calibration curve, a standard solution of catechin was used (range 20-1000 mgL^{-1} in methanol). An aliquot of extract (1 mL) was mixed with 2.5 mL of 1% vanillin solution (in methanol) and 2.5 mL of 9M hydrochloric acid. After incubation for 20 minutes at 30°C, absorbances were measured at 500 nm, and A was calculated, using equation 1.

$$\mathrm{A} = (A_s - A_b) - (A_c - A_0) \qquad (1)$$

A_s - absorbance of the sample (1 mL of catechin solution or extract + 2.5 mL of 1% vanillin solution + 2.5 mL of 9M hydrochloric acid)

A_b- absorbance of the sample with 0 mg of catechin (1 mL of water + 2.5 mL of 1% vanillin solution + 2.5 mL of 9M hydrochloric acid)

A_c – absorbance of the control (1 mL of catechin solution or extract + 2.5 mL of methanol + 2.5 mL of 9M hydrochloric acid)

A_0 – absorbance of the control with 0 mg of catechin (1 mL of water + 2.5 mL of methanol + 2.5 mL of 9M hydrochloric acid)

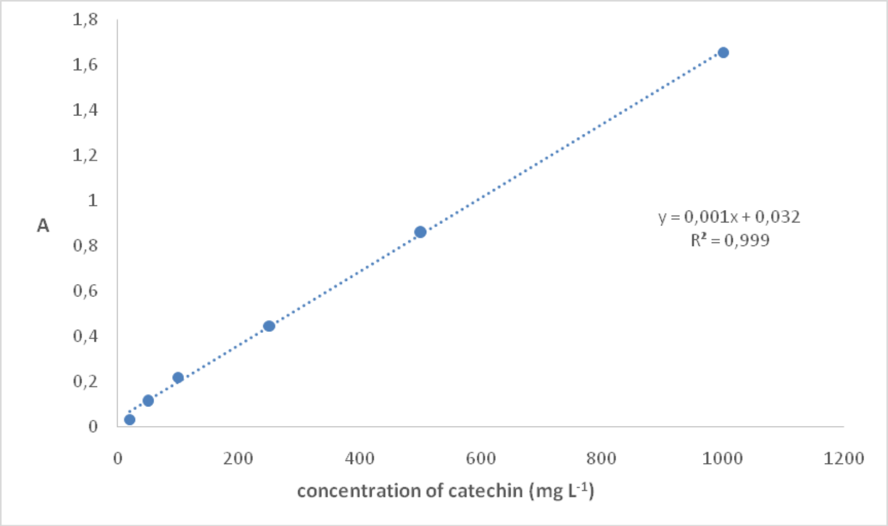

SUMMARY OUTPUT						
Regression Statistics						
Multiple R	0,999514019					
R Square	0,999028275					
Adjusted R Square	0,998785344					
Standard Error	0,021407095					
Observations	6					
ANOVA						
	df	*SS*	*MS*	*F*	*Significance F*	
Regression	1	1,884559778	1,88456	4112,39146	3,54208E-07	
Residual	4	0,001833055	0,000458			
Total	5	1,886392833				
	Coefficients	*Standard Error*	*t Stat*	*P-value*	*Lower 95%*	*Upper 95%*
Intercept	0,03285443	0,011932207	2,753424	0,05119401	-0,00027469	0,065983549
X Variable 1	0,001628059	2,53877E-05	64,12793	3,5421E-07	0,001557572	0,001698547

Figure S3. Calibration curve for the determination of the proanthocyanidin content

Total anthocyanins content calculation

For the calculation of the content of anthocyanins (expressed as cyanidin-3-glucoside equivalents, mgL^{-1}) equation 2 was used:

$$\text{Anthocyanins} = \frac{A \cdot MW \cdot DF \cdot 1000}{\varepsilon \cdot l} \qquad (2)$$

Where, A = $(A_{520nm} - A_{700nm})_{pH1.0} - (A_{520nm} - A_{700nm})_{pH\ 4.5}$; MW = molecular weight of cyanidin-3-glucoside (449.2 g mol^{-1}); DF = dilution factor; ε = molar extinction coefficient (26 900 L mol $^{-1}$cm^{-1}); l = optical pathlenght (1 cm); 1000 - factor for conversion (g to mg).

DPPH (2,2-Diphenyl-1-picrylhydrazyl) assay calculation

$$\%\ \text{of inhibition} = \frac{A_{c.s.(0s)} - A_{s(960s)}}{A_{c.s.(0s)}} \cdot 100 \qquad (3)$$

$A_{c.s.(os)}$= absorbance of the control solution (blank) measured after 0 seconds

$A_{s(960s)}$= absorbance of the sample (extract) measured after 960 seconds

FRAP (Ferric Ion Reducing Antioxidant Power) assay

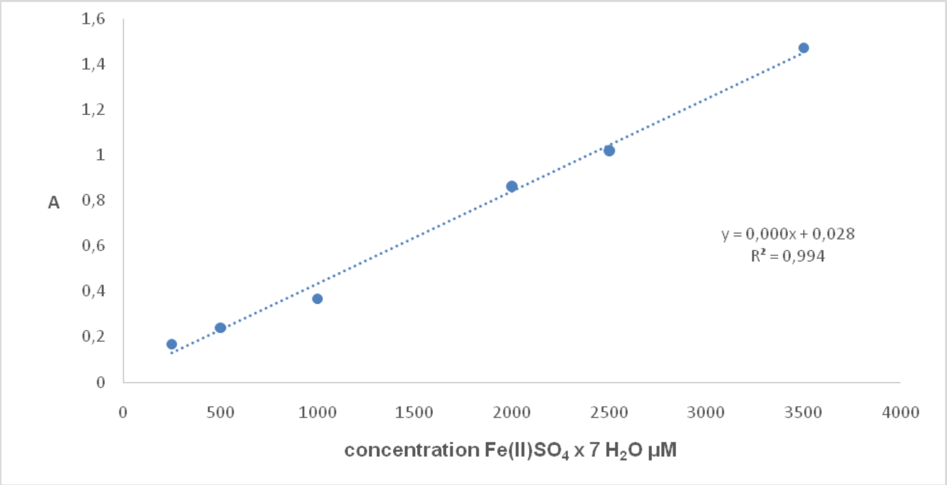

SUMMARY OUTPUT

Regression Statistics	
Multiple R	0,99712305
R Square	0,99425438
Adjusted R Squar	0,99281797
Standard Error	0,04357493
Observations	6

ANOVA

	df	*SS*	*MS*	*F*	*Significance F*
Regression	1	1,314298235	1,314298	692,1824	1,24033E-05
Residual	4	0,007595098	0,001899		
Total	5	1,321893333			

	Coefficients	*Standard Error*	*t Stat*	*P-value*	*Lower 95%*	*Upper 95%*
Intercept	0,02839216	0,030751653	0,923273	0,408118	-0,05698812	0,113772434
X Variable 1	0,00040612	1,54362E-05	26,30936	1,24E-05	0,00036326	0,000448976

Figure S4. Calibration curve for the FRAP assay

Total antioxidant capacity (TAC)

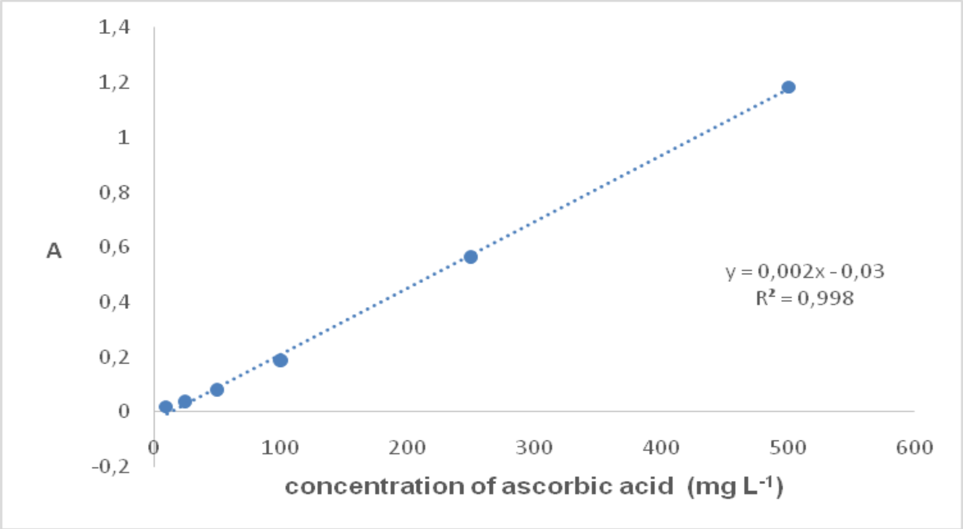

SUMMARY OUTPUT						
Regression Statistics						
Multiple R	0,999304093					
R Square	0,99860867					
Adjusted R Square	0,998260838					
Standard Error	0,019070784					
Observations	6					
ANOVA						
	df	*SS*	*MS*	*F*	*Significance F*	
Regression	1	1,044148554	1,044149	2870,947	7,26262E-07	
Residual	4	0,001454779	0,000364			
Total	5	1,045603333				
	Coefficients	*Standard Error*	*t Stat*	*P-value*	*Lower 95%*	*Upper 95%*
Intercept	-0,02996801	0,010472662	-2,86155	0,045858	-0,059044784	-0,0008912
X Variable 1	0,002408351	4,49477E-05	53,58122	7,26E-07	0,002283556	0,0025331

Figure S5. Calibration curve for the determination of total antioxidant capacity (TAC)

ABTS values calculation

ABTS values expressed as Trolox equivalent (TE, μmol Trolox L^{-1}) were calculated using equation 4.

$$\text{TEAC} = 3 \cdot 51 \cdot \frac{AUC_{sample}}{\text{r.c. Trolox}} / 1000 \qquad (4)$$

where 3 is a dilution factor for the Trolox, 51 is a dilution factor for the sample, and r.c.$_{\text{Trolox}}$ is a regression coefficient calculated from the calibration curve (equation 5.)

$$\text{AUC}_{\text{Trolox}} = r.c._{Trolox} \cdot [Trolox] \qquad (5)$$

AUC$_{\text{sample}}$ and AUC$_{\text{Trolox}}$ were calculated using equation 6.

$$\text{AUC} = (\%inh_{(t=0)} \cdot 0.5 + \sum_{i=1}^{90} \%inh_{(t=10i)}) \cdot 10 \qquad (6)$$

Where the % inh$_{(t)}$ describes percentage of inhibition in t-seconds.

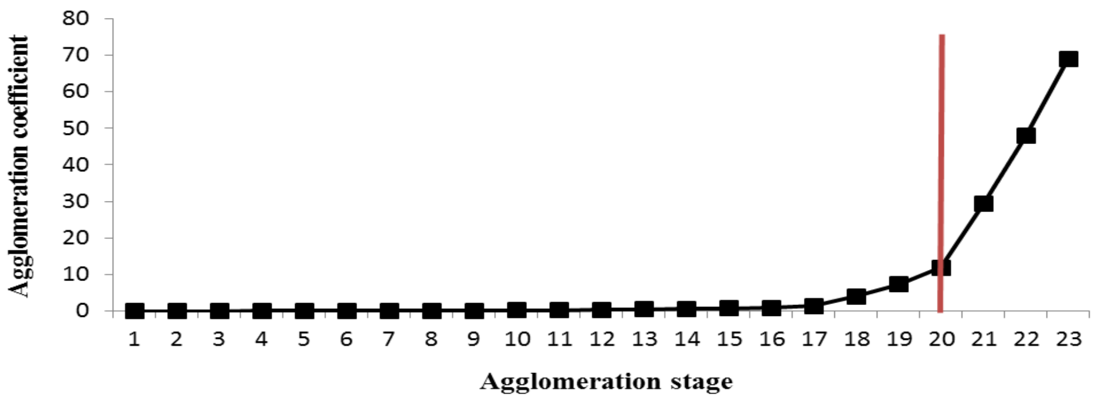

Figure S6. The plot of agglomeration coefficients generated by Ward's hierarchical clustering analysis

Table S1. Agglomeration schedule table generated by the Ward's chierarchical clustering analysis

Stage	Cluster Combined		Coefficients	Stage Cluster First Appears		Next Stage
	Cluster 1	Cluster 2		Cluster 1	Cluster 2	
1	23	24	0.003	0	0	7
2	3	4	0.008	0	0	11
3	15	16	0.012	0	0	15
4	5	7	0.017	0	0	8
5	9	10	0.024	0	0	12
6	18	19	0.049	0	0	14
7	1	23	0.074	0	1	18
8	5	6	0.103	4	0	18
9	11	12	0.134	0	0	16
10	21	22	0.164	0	0	13
11	2	3	0.208	0	2	17
12	8	9	0.324	0	5	20
13	20	21	0.450	0	10	20
14	17	18	0.585	0	6	23
15	14	15	0.744	0	3	17
16	11	13	0.930	9	0	19
17	2	14	1.399	11	15	19
18	1	5	3.974	7	8	22
19	2	11	7.297	17	16	21
**20***	8	20	**11.847**	12	13	21
21	2	8	29.360	19	20	22
22	1	2	47.918	18	21	23
23	1	17	69.000	22	14	0

* Stage at wich the lagest increase in value is observed (the „elbow“)

Table S2. Cluster membership determined by *k*-means clustering analysis

Case number	Sample name	Cluster	Distance
1	serviceberry 3	1	0.764
2	red currant 1	2	0.745
3	red currant 2	2	0.632
4	red currant 3	2	0.565
5	gooseberry 1	1	0.755
6	gooseberry 2	1	0.546
7	gooseberry 3	1	0.673
8	bilberry 1	3	1.191
9	bilberry 2	2	1.292
10	bilberry 3	2	1.191
11	cornelian cherry 1	2	1.024
12	cornelian cherry 2	2	0.957
13	cornelian cherry 3	2	0.763
14	black currant 1	2	0.748
15	black currant 2	2	0.470
16	black currant 3	2	0.549
17	black chokeberry 1	4	0.300
18	black chokeberry 2	4	0.057
19	black chokeberry 3	4	0.258
20	blackberry 1	3	0.172
21	blackberry 2	3	0.496
22	blackberry 3	3	0.595
23	serviceberry 1	1	0.578
24	serviceberry 2	1	0.631
